# Flavonoids: a metabolic network mediating plants adaptation to their real estate

**DOI:** 10.3389/fpls.2014.00620

**Published:** 2014-11-10

**Authors:** Aidyn Mouradov, German Spangenberg

**Affiliations:** ^1^Royal Melbourne Institute of Technology UniversityBundoora, VIC, Australia; ^2^Department of Environment and Primary Industries, Biosciences Research Division, AgriBio, Centre for AgriBioscienceBundoora, VIC, Australia; ^3^School of Applied Systems Biology, La Trobe University – AgriBio, Centre for AgriBioscienceBundoora, VIC, Australia

**Keywords:** flavonoids, anthocyanins, proanthocyanidins, evolution, transgenic, metabolism

## Abstract

From an evolutionary perspective, the emergence of the sophisticated chemical scaffolds of flavonoid molecules represents a key step in the colonization of Earth’s terrestrial environment by vascular plants nearly 500 million years ago. The subsequent evolution of flavonoids through recruitment and modification of ancestors involved in primary metabolism has allowed vascular plants to cope with pathogen invasion and damaging UV light. The functional properties of flavonoids as a unique combination of different classes of compounds vary significantly depending on the demands of their local real estate. Apart from geographical location, the composition of flavonoids is largely dependent on the plant species, their developmental stage, tissue type, subcellular localization, and key ecological influences of both biotic and abiotic origin. Molecular and metabolic cross-talk between flavonoid and other pathways as a result of the re-direction of intermediate molecules have been well investigated. This metabolic plasticity is a key factor in plant adaptive strength and is of paramount importance for early land plants adaptation to their local ecosystems. In human and animal health the biological and pharmacological activities of flavonoids have been investigated in great depth and have shown a wide range of anti-inflammatory, anti-oxidant, anti-microbial, and anti-cancer properties. In this paper we review the application of advanced gene technologies for targeted reprogramming of the flavonoid pathway in plants to understand its molecular functions and explore opportunities for major improvements in forage plants enhancing animal health and production.

## INTRODUCTION

The multicellular algae that changed their marine environment to the harsh terrestrial had to face numerous challenges including: higher oxygen concentration, desiccation, increasing gravity, damaging heat and UV light, greater diurnal and seasonal fluctuations in temperatures, chances to be infected and eaten by new pathogens and grazers. Decision to stand upright spreading branches horizontally to catch more sun light will require an efficient strategy to fight against gravity and establish a system of waterproof ‘pipes’ pumping water from the roots to the rest of the plants. New inventive strategy recruiting insects for pollination will need development of the first colorful flowers and volatile chemicals. A seed dispersal by first representatives of moving, running and flying vegetarian dinosaurs will associate with an urgent demand for quick-reacting machinery to adapt to new geographical locates. And the fact that plants had to overcome all of these challenges standing on the same place made their evolution perspective a ‘mission impossible.’

Today, more than 500 million years later, we observe that not only all of these challenges were successfully overcome, but modern plants represent most evolutionally successful eukaryotic species colonizing all parts of our planet, between North and South poles. If the development of unique stem cells machinery contributed to plants’ longevity (some trees can live for 100s and 1000 years, when just few animals reach their 100th birthday; Weigel and Jurgens, 2002) it is the evolution of plant-specialized secondary metabolites which made their life ‘safe and comfortable’ in new established real estates (Winkel-Shirley, 2001a, 2002; Agati et al., 2012; Ferreyra et al., 2012a; Saito et al., 2013). The acquisition of the secondary metabolites including flavonoids represents an important step in the colonization of Earth’s terrestrial environment by vascular plants. Physiological functions of flavonoids and the reasons for their ubiquitous existence have been widely discussed. A short list of these functions includes: (i) protection against insect predation and defense against microbes (Kliebenstein, 2004; Bidart-Bouzat and Kliebenstein, 2008); (ii) action as sunscreens to absorb UV radiation and strong light, thus replacing mycosporine-like amino acids usually detected in algae (Guo et al., 2008b; Agati et al., 2013); (iii) attraction of insect pollinators through production of colorful anthocyanins, absorbing different spectra of visible light (Winkel-Shirley, 2001a,b); (iv) action as antioxidants, inhibiting the generation of reactive oxygen species (ROS), by maintaining their concentration within a sub-lethal range (Blokhina et al., 2003; Agati et al., 2012, 2013; Stolarzewicz et al., 2013); (v) involvement in pollen germination (Tanaka and Ohmiya, 2008; Tanaka et al., 2008; Ferreyra et al., 2012b); (vi) involvement in biological communication in the rhizosphere (Cesco et al., 2012; Weston and Mathesius, 2013) and action as (vii) developmental regulators, involved in auxin transport and catabolism (Friml and Jones, 2010).

## EVOLUTION OF FLAVONOIDS

Our understanding of the evolution of flavonoid pathway has considerably increased over the last decade. However, the remaining ‘gaps’ in our understanding of the primary evolutionary roles of flavonoids in early land plants can be explained by the fact that modern plants might differ significantly from ancestors in their priorities for flavonoid’s functions. It’s been widely accepted that elavonoid molecules are a unique ‘invention’ of land plants assuming that algal and cyanobacterial cells are and were ‘flavonoids-free’ (Iwashina, 2000; Markham, 2006; Rausher, 2006). However, first a study reported that representatives of streptophyte Nitella, the most closely related algae to terrestrial plants, also contains flavonoids was published in 1969 (Markham and Porter, 1969). Later, the phenylpropanoid pathway enzyme, phenylalanine ammonia lyase (PAL) that catalyzes the first and essential step of the general phenylpropanoid pathway, was detected in the cyanobacteria *Anabaena variabilis* and *Nostoc punctiforme* (Moffitt et al., 2007) and in the green microalga *Chlorella pyrenoidosa* (Chen et al., 2003). Recent extensive algal genomes sequencing program revealed a number of homologs of plant flavonoid genes including, *chalcone isomerase* (CHI) and *isoflavone reductase* in *Chlamydomonas* (May et al., 2008), *dihydrokaempferol-4-reductase* and *naringenin chalcone synthase* (CHS) in *Phaeodactylum* (Bowler et al., 2008), and *CHI* and *dihydroflavonol reductase* in *Ectocarpus* (Cock et al., 2010). It was, however, suggested that some of these enzymes could be involved in other than flavonoid pathways, for example, fatty acid biosynthesis (Ngaki et al., 2012). Breakthrough in this area was recently made by Goiris et al. (2014), who analyzed all known precursors, key intermediates and end products of the flavonoid biosynthetic pathway in representatives of divergent algal lineages (*Cyanobacteria, Rhodophyta, Chlorophyta, Haptophyta, Ochrophyta*). This research showed that distant microalgae representatives contain a wide range of flavonoids which composition is compatible with the established basic flavonoid pathway observed in higher plants. This implies that the flavonoid biosynthetic pathway arose much earlier in evolution compared to what is generally accepted and could belong to the phylum Plantae, which involves plants, glaucocystophytes, red algae, and green algae (Baldauf, 2003).

Evolutionary, small quantities of flavonoids may be existed in early representatives of algae and plants as side products of enzymes of primary metabolism as a result of the proximity of their substrate binding domains. Stepwise and lengthy development of first flavonoids was a result of a number of evolutionary processes which led to production of first three enzymes, CHS, CHI, and flavanone 3-hydroxylase (F3H) in representatives of bryophytes (mosses), liverworts, and hornworts the earliest plants colonizing Earth (Markham, 1988; Verwoert et al., 1992; Winkel-Shirley, 2001a). Some of these enzymes could evolve via gene duplication of genes of primary metabolism. Alternatively, key phenylpropanoid enzymes could be acquired via horizontal gene transfer during plant symbioses with bacteria and fungi that are known to be established very early during the first steps of land colonization. It was suggested that PAL was horizontally acquired from PAL homologs existed in sediment/soil bacteria (Emiliani et al., 2009). CHI-like enzymes were also present in fungi, early branching mycetozoa, and in some representatives of proteobacteria and could also be acquired via horizontal gene transfer (Gensheimer and Mushegian, 2004). Evolution of *CHS* and *F3H* was a result of recruitment and gene duplication of representatives of polyketide synthases and the oxoglutarate-dependent dioxygenase, respectively, from primary metabolism. Activities of the CHS, CHI, and F3H led to production of the first three flavonoids, chalcones, flavonols, and flavones which represent the oldest plant flavonoids. These metabolites, remained intact over 500 million years, represent core intermediates for modern pathways producing a massive spectrum flavonoid products with irreducible complexity.

Two main hypotheses regarding the primary roles of flavonoids proposed that these molecules evolved (i) as an effective sunscreen protecting against UV radiation as plants began colonizing land (Markham, 1988) or (ii) as developmental regulators of auxin transport and catabolism (Stafford, 1991). Protection from damaging UV light as a presumable primary function of ancestral flavonoids does not match well with the observation that representatives of flavonols (quercetin), produced in early land plants do not absorb UV-B wavelengths as efficiently as other flavonoids and phenolics (Harborne and Williams, 2000). Moreover, these flavonols have much lesser ability to absorb light wavelengths over the 290–320 nm and UV-B than their ‘counterparts’ from ancestral algae, mycosporine like aminoacid (MAA; Harborne and Williams, 2000; Agati et al., 2012, 2013). And, since flavonoids exist at nanomolar concentrations it is unlikely they could be immediately effective as an efficient sunscreen from damaging UV penetration into leaf tissues. Moreover, recent research on role of flavonoids in photoprotection showed that flavonoids accumulate not only in epidermal cells but also in the mesophyll cells after exposure to sunlight (Agati et al., 2012, 2013).

Involvement of flavonoids in nanomolar concentrations as signal molecules mediating the cascades of oxidative stresses and as regulators of intra-cellular and long-distance movements of multifunctional growth regulators, such as auxins is well investigated (Taylor and Grotewold, 2005; Peer and Murphy, 2007; Wenzel et al., 2008; Friml and Jones, 2010). In modern plants quercetin, kaempferol, apigenin were found to be involved as signaling molecules in auxin-regulated cell division (Peer and Murphy, 2007; Buer and Djordjevic, 2009; Kuhn et al., 2011; Lewis et al., 2011). Mutation of flavonoids can trigger pleiotropic effect on overall plants architecture affecting root growth, lateral root density, root hair development and length, shoot/flower organ number, and seed organ density and fertility (Harborne and Williams, 2000; Buer and Djordjevic, 2009; Agati et al., 2012, 2013). It was also recently shown that activated by light auxin can regulate the glycosylation pattern of different flavonoids in response to UV-B significantly contributing to stress-induced morphogenetic responses (Hectors et al., 2012).

Nuclear localization of CHS and CHI enzymes (Saslowsky and Winkel-Shirley, 2001; Saslowsky et al., 2005) and also flavonoids, their interaction with mitotic chromosomes and histone proteins, regulation of chromatin conformation, involvement in epigenetic processes and gene expression attract increasing attention among wide spectrum of scientists (Feucht et al., 2004, 2012, 2013; Taylor and Grotewold, 2005). Anti-oxidative role of nuclear flavonoids protects mitotic chromosomes at stages when they are not enveloped by nuclear membrane and are most sensitive to oxidative stresses (Feucht et al., 2012). Fragmentation of DNA was shown be markedly reduced by monomeric and dimeric catechins through donation of electrons or hydrogens to transcriptionally important basic amino acids of histones, such as lysine, arginine, and histidine, protecting them against oxidative stress (Huang et al., 2006; Stangl et al., 2006a,b; Guo et al., 2008a). Binding of flavonoids to the histone and none-histone chromatin binding proteins can change chromatin’s conformation affecting gene expression (Bidel et al., 2010). Changes in concentrations of nuclear flavonoids were observed in response to environmental stresses, such as heat and drought (Feucht et al., 2013).

Accumulated data suggest that the oxidative stress-induced signaling and regulatory functions of flavonoids very likely represent their primary roles in early plants. Most of these functions has remained intact over 500 million years of evolution and are conserved across the kingdoms of modern plants. Other functions such as the direct protection of plants from environmental stresses such as UV sunscreen, pigmentation and others were gradually evolved at later stages when concentrations and spectrum of flavonoids were increased and reached levels observed in modern terrestrial plants (Ferrer et al., 2008).

The stepwise emergence, diversity and evolutionary success of flavonoid pathway were triggered by a number of major events: (i) recruitment of enzymes from primary metabolism including the shikimate, the phenylpropanoid and polyketide pathways through the cascades of gene duplication events; (ii) horizontal gene transfer during plant/algal symbioses with bacteria and fungi; (iii) modification of the metabolic enzymes through the changes in regiospecificity (ability to modify different parts of the substrate molecules) and substrate selectivity (ability to bind to different substrates); (iv) the changes in regulatory control of flavonoid genes; (v) plasticity of flavonoid pathways and their ability depending on the demands of the local ecosystem to redirect the flows of intermediate molecules towards biosynthesis of the sophisticated scaffolds of very different chemicals. As a result of these evolutionary events more than 10,000 of these chemicals have been identified in more than 9000 modern plant species making flavonoids one of the most widely spread pathways in modern plants (Winkel-Shirley, 2001a,b).

Decades of study of the flavonoid, anthocyanin, proanthocyanidin and lignin pathways have coalesced around understandings of molecular aspects of some of these events including: (i) detailed gene-to-metabolite study of above mentioned metabolic pathways linking spatio-temporal profiles of their gene expression to the functional metabolomics of related products and (ii) analysis of the molecular and metabolic cross-talks between the flavonoid and other pathways in response to stresses.

## PLASTICITY OF FLAVONOID PATHWAY

### MOLECULAR ASPECTS OF FLAVONOID PATHWAY

**FIGURE 1:**
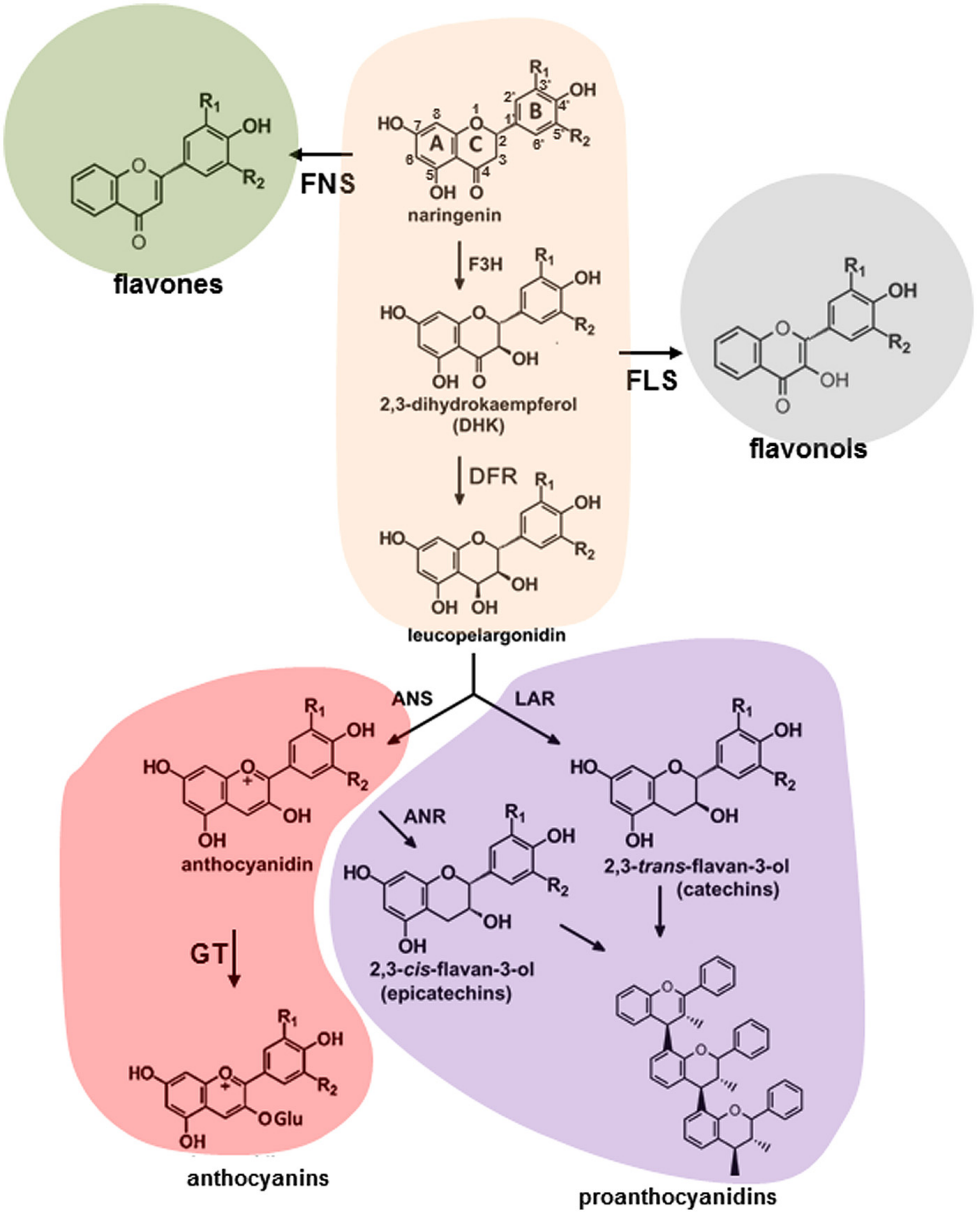
**Schematic representation of flavonoid pathway in plants.** ANR, anthocyanidin reductase; ANS, anthocyanidin synthase; DFR, dihydroflavonol 4-reductase; F3H, flavonoid-3′ hydroxylase; FLS, flavonole synthase; FNS, flavone synthase; GT, glucosyltransferase; LAR, leucoanthocyanidin reductase.

Molecular aspects of flavonoid pathway were extensively investigated and reviewed (Winkel-Shirley, 2001a,b; Grotewold, 2006a; Saito et al., 2013; Doughty et al., 2014). Plant phenolics are products of the shikimate, phenylpropanoid, flavonoid, anthocyanin, and lignin pathways. The shikimate pathway produces the aromatic amino acids, including phenylalanine, which can be further modified through the sequential of elongation and cyclization steps to form the flavonoids (**Figure 1**). In general, flavonoids are sub-classified into several families of fifteen-carbon molecules including flavonol, flavone, flavanone, flavan-3-ol, isoflavone, and anthocyanidin (Ferreyra et al., 2012b). As a first step, phenylalanine gets converted into coumaroyl-CoA by a number of enzymatic reactions involving PAL, cinnamate 4-hydroxylase (C4H) and 4-coumarate:CoA ligase (4CL). The CHS and CHI convert coumaroyl-CoA into naringenin and onward, the pathway diverges forming flavanones, dihydroflavonols, leucoanthocyanins, anthocyanidins, and flavan-3-ols through a series of enzymatic steps shown in **Figure 1**. These molecules can produce a series of products, flavones and isoflavones, flavonols, anthocyanins, and proanthocyanidins (condensed tannins) existing in the forms of monomers, dimers, and polymers (Chan et al., 2009; Pati et al., 2009; Abeynayake et al., 2012; Hancock et al., 2014). Three main classes within these molecules are differing only in the extent of B-ring hydroxylation (Abrahams et al., 2002; Pang et al., 2007; Tanaka et al., 2008).

Cell-specific and intracellular localization of flavonoids has indeed been extensively investigated. Flavonoids were found in epidermal cells, including trichomes as well as within palisade and spongy mesophyll cells (Agati et al., 2002; Schmitz-Hoerner and Weissenbock, 2003; Tattini et al., 2005). Intracellularly flavonoids also occur in various cell compartments such as chloroplasts, vacuole and nucleus (Polster et al., 2006; Mukai et al., 2009; Abeynayake et al., 2011, 2012, 2014; Agati et al., 2012, 2013).

### CROSS-TALK BETWEEN ANTHOCYANIN AND PROANTHOCYANIDIN PATHWAYS

Despite the fact that anthocyanin and proanthocyanidin pathways share the same metabolic intermediates they represent the most and the least extensively investigated branches of flavonoid pathway, respectively. Both branches involve the conversion of 4-coumaroyl-CoA and malonyl-CoA to the families of precursors (**Figure 1**; Winkel-Shirley, 2001a,b; Grotewold, 2006a; Abeynayake et al., 2012). Dihydroflavonol 4-reductase (DFR), anthocyanidin synthase (ANS), as well as a range of anthocyanidin-modifying enzymes convert dihydrokaempferols, dihydroquercetins, and dihydromyricetins into anthocyanins with pelargonidin cyanidin and delphinidin backbones, respectively (Tanaka and Ohmiya, 2008). A list of anthocyanidin-modifying enzymes includes glycosyltransferases, UDP-glucuronosyl/UDP-glycosyltransferases, glutathione transferases, methyltransferases, and anthocyanidin rhamnosyltransferases. In proanthocyanidin-specific pathway dihydroflavonols are converted to *cis-* and *trans*-epimeric forms of afzelechins, catechins, and gallocatechins, GCs by the family of DFR, leucoanthocyanidin reductase (LAR), ANS, and anthocyanidin reductase (ANR) enzymes.

**FIGURE 2:**
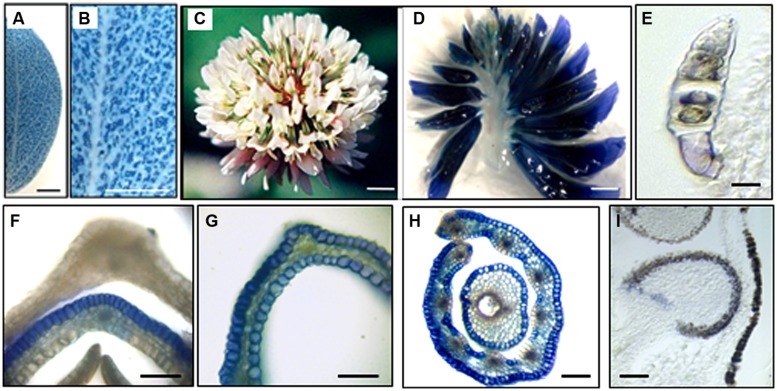
**Accumulation of proanthocyanidins in *Lotus corniculatus* and *Trifolium repens* organs.** Abeynayake et al. (2011,^2012^). **(A,B)** *L. corniculatus* leaf stained with DMACA; **(C)** mature *T. repens* inflorescence; **(D)** longitudinal section through mature *T. repens* inflorescence stained with DMACA; **(E)** longitudinal section through a trichome; **(F)** transverse section through an immature petal in which proanthocyanidins accumulated only on the abaxial side. **(G)** transverse section through a mature standard petal showing the accumulation of proanthocyanidins on both the abaxial and adaxial sides; **(H)** transverse section through anther filaments and carpel; **(I)** longitudinal section through a developing seed in a mature flower. Bars = 200 μm **(A,B)**; 1 mm **(C,D)**; 5 μm **(E)**; 50 μm **(F,G)**, 100 μm **(G)**, and 10 μm **(I)**.

Proanthocyanidins, polymers of flavan-3-ol subunits are best known for their protein-binding ability and are commercially significant because of their antioxidant properties and their potential health benefits when included at a low level in the diets of humans and livestock (McNabb et al., 1996; Wang et al., 1996b; Yu et al., 1996; Waghorn and McNabb, 2003; Hancock et al., 2014). Proanthocyanidins are found in the leaves, flowers, fruit, seeds, bark, and roots of many plant species (Abeynayake et al., 2011, 2012; Hancock et al., 2014). Legumes offer opportunities for studying proanthocyanidin biosynthesis accumulating them across a broad range of tissues [for reviews, see (Marles et al., 2003; Lepiniec et al., 2006; Routaboul et al., 2006]. *Medicago sativa* and *M. truncatula* accumulate a high level of proanthocyanidins in seed coats, but low levels in flowers, stems, roots, and leaves (Pang et al., 2007). Members of *Lotus* species accumulate to the large extent proanthocyanidins in their leaf mesophyll cells (**Figure 2**; Abeynayake et al., 2011), which makes them very attractive for ruminants health and production (Escaray et al., 2012, 2014). White clover (*Trifolium repens*), a major component of temperate pastures worldwide accumulates proanthocyanidins mainly in floral organs and seeds (**Figure 2**; Abeynayake et al., 2011, 2012). Accumulation of proanthocyanidins in vegetative tissues (leaves and stems) is restricted to the trichome cells.

**FIGURE 3:**
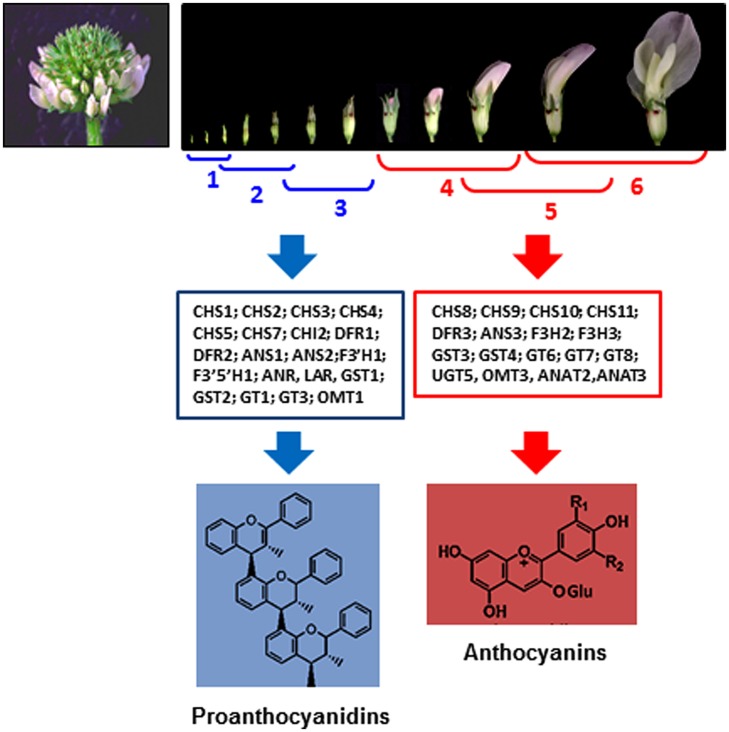
**Developmentally regulated biosynthesis of proanthocyanidins and anthocyanins in white clover flowers (Abeynayake et al., 2012).** Upper figure shows six stages of flower development in white clove. Middle figure shows flavonoid gene families and gene members related to the biosynthesis of proanthocyanidins and anthocyanins, differentially expressed at stages 1–3 and 4–6, respectively.

In white clover flowers the epidermal cells of petals and inner organs, such as stamen filaments, carpels, ovules, and immature embryos were intensively investigated by our group because of spatial co-localization of the anthocyanin and proanthocyanidin pathways (Abeynayake et al., 2011, 2012). Biosynthesis of proanthocyanidins in epidermal cells of these organs starts in cells located on adaxial side followed by their production on abaxial sides (**Figure 2**). Accumulation of the 2,3-*trans*-flavan 3-ols (gallocatechins, GC) and 2,3-*cis*-flavan 3-ols (epigallocatechins, EGC), the building blocks of proanthocyanidins is developmentally regulated being active at the early stages of flowers development (stages 1–3) followed by their sharp down-regulation at the later stages of development (stages 4–6; **Figure 3**; Abeynayake et al., 2012). This suggests that in floral organs biosynthesis of proanthocyanidins is not light-regulated and is active at the stages when proanthocyanidin-producing organs are well protected from light by sepals. Exposure of the petals to light at the stages 4–6 correlates with significant increase of glycosylated forms of flavonols, representing all three B-ring hydroxylated variants, with an abundance of myricetin glycosides (m/z 479; R3# = OH, R5# = OH) and quercetin glycosides (m/z 463; m/z 505; R3# = OH, R5# = H) and the onset of light-induced anthocyanins with a predominance of delphinidins is synthesized. A low level of kaempferol-based anthocyanins (m/z 447; R3# = H, R5# = H) and virtually no pelargonidin-based anthocyanins were found in flowers. Developmentally regulated profiles of proanthocyanidin and anthocyanin biosynthesis were confirmed by developmentally regulated profiles of expression of known flavonoid-related genes. Generated gene-to-metabolite data suggest that these pathways, are potentially recruiting different isoforms of the same flavonoid biosynthetic enzymes, represented by distinct members of multigene families (**Figure 3**).

Cross talk between members of proanthocyanidin- and anthocyanin-specific branches of the flavonoid pathways was shown in a number of plant species. Ectopic expression of *ANR* gene in tobacco down-regulated anthocyanin production and resulted in the biosynthesis of proanthocyanidins in flower petals (Xie et al., 2003). Ectopic expression of *ANR* in *M. truncatula* plants resulted in a decrease of approximately 50% in anthocyanin production and up to a threefold increase in proanthocyanidin production in a subset of leaf cells (Xie et al., 2006). Re-direction of anthocyanin pathway into proanthocyanidin pathway in *Nicotiana tabacum, M. sativa*, and *T. repens* plants was achieved by ectopic expression of *TaMYB14* from *T. arvense* (Hancock et al., 2012). In white clover plants, expression of *TaMYB14* led to accumulation of proanthocyanidins in leaves up to 1.8% dry matter. Silencing of *TaMYB14* resulted in almost complete cessation of proanthocyanidin biosynthesis in *T. arvense* (Hancock et al., 2014). Production of commercial white clover cultivars with enhanced levels of foliar condensed tannins could significantly contribute to pasture productivity via increased production, forage quality and decreases in adverse environmental impacts.

**FIGURE 4:**
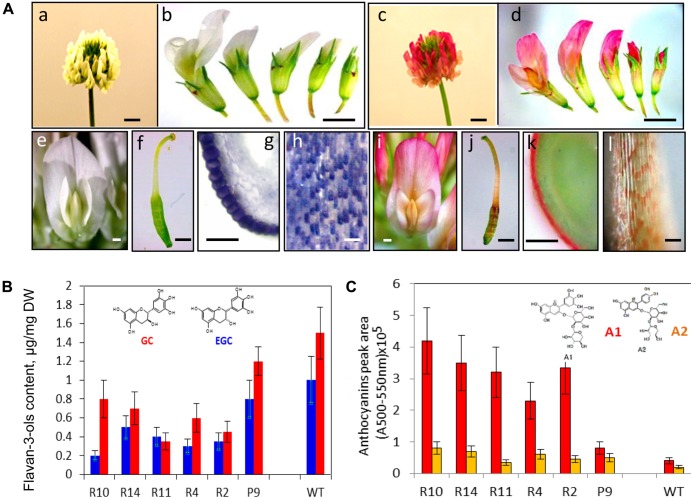
**Phenotypes **(A)** and biochemical profiles **(B)** of TrANRhp transgenic white clover lines with down-regulated *TrANR* gene (Abeynayake et al., 2012). (A)**: (a) wild-type white clover inflorescence; (b) wild-type flowers at different developmental stages; (c) TrANRhp inflorescence; (d) TrANRhp flowers at different developmental stages; (e) wild-type mature flower; (f) wild-type carpel; (g) cross-section of a carpel stained with DMACA; (h) epidermal cells of anther filaments stained with DMACA; (i) TrANRhp mature flower; (j) TrANRhp carpel; (k) cross-section of TrANRhp carpel; (l) epidermal cells of TrANRhp anther filaments. **(B)** level and composition of flavan 3-ols; **(C)** level and composition of anthocyanins. Bars = 2 mm (a–d); 1 mm (e,f,i,j); 500 μm (g,k) and 75 μm (h,l). GC, gallocatechin; EGC, epigallocatechin; A1, delphinidin 3-sambubioside; A2, cyanidin 3-sambubioside.

Redirection of flavonoid biosynthesis from proanthocyanidin into anthocyanin biosynthesis was firstly shown as production of red-colored seed coats in the *Arabidopsis banyuls* mutant (Xie et al., 2003). Down-regulation of *ANR* in this mutant led to the precocious accumulation of anthocyanins in proanthocyanidin-producing tissues (Devic et al., 1999; Abrahams et al., 2003; Xie et al., 2003). Down-regulation of the *TrANR* gene in white clover plants and associated reducing level of EGCs have triggered changes in accumulation profiles of delphinidin-based anthocyanins as a result of the diversion of intermediates from flavan 3-ol to anthocyanin production (**Figure 4**; Abeynayake et al., 2012). This triggered accumulation of anthocyanins in all epidermal cells which normally produced proanthocyanidins in wild-type plants (**Figure 4**). Moreover, light-independent biosynthesis of anthocyanins in red-flowered TrANRhp lines is developmentally regulated, with most intense red coloration produced at the stage 3 when proanthocyanidin pathway is most active in wild-type flowers. Metabolic redirection of intermediate molecules in these plants is associated with significant changes in expression profiles of the late flavonoid genes involved in anthocyanin production, the members of the glycosyltransferases, UDP-glucuronosyl/UDP-glycosyltransferases, glutathione transferases, methyltransferases, and anthocyanidin rhamnosyltransferases. The most striking results of this experiment was the fact that down-regulation of the *ANR* gene has triggered significant changes in level and profiles of early flavonoids which biosynthesis occurs in the upstream part of flavonoid pathway (Abeynayake et al., 2012). This can be explained by changes in the expression profiles of the gene families functioning upstream of *TrANR*. A list of affected upstream genes includes members of the early flavonoid pathway namely *CHS, CHI, F3H, DFR, ANS, CHR* as well as genes involved in isoflavone biosynthesis, *IF3#H-, IFOMT-,* and *VR-like* genes. Global changes in expression profiles of flavonoid genes associated with up- and down-regulation of the members of transcription factors in TrANRhp lines is providing further support for transcriptional regulation of this pathway (Endt et al., 2002; Johnson et al., 2002; Nesi et al., 2002; Dubos et al., 2008; Hichri et al., 2011; Petroni and Tonelli, 2011).

### CROSS-TALK BETWEEN LIGNIN AND FLAVONOID PATHWAYS

The emergence and evolution of chemical scaffolds of lignin polymers provided plants with mechanical support, protected them from pathogen invasion, damaging UV, and enhanced the hydrophobicity of their vasculature. Thus these metabolites have been of paramount importance for the evolution of land plants and their colonization of the distinct geographical and ecological locales. Similar to flavonoids also this pathway recruited enzymes from primary metabolism to promote the biosynthesis of H and G lignin in early terrestrial plants. Evolutional advantage of S lignin in adaptation to environment was a result of the selective structural alterations of the ring modification enzymes such as ferulate 5-hydroxylase and caffeic acid/5-hydroxyferulic acid *O*-methyltransferase (COMT) at later stages of evolution (Weng and Chapple, 2010). Lignification consequently transformed phenylpropanoid metabolism into a major sink for carbon in plants estimated to represent as much as 30% of the total biomass produced in the biosphere (Boerjan et al., 2003).

**FIGURE 5:**
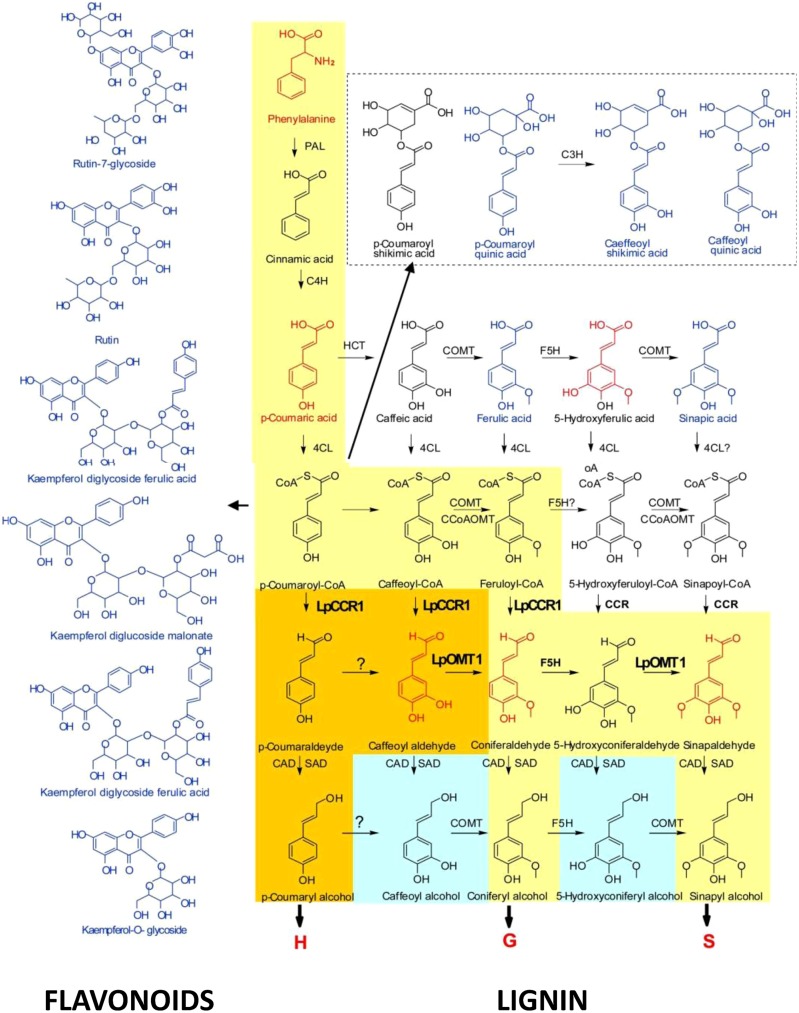
**Cross-talk between monolignol and flavonoid pathways ( Tu et al., 2010).** The yellow route toward the production of monolignols is conserved in angiosperms. The orange route is found in perennial ryegrass. The blue route is found in some species. CAD, cannery alcohol dehydrogenase; 4CL, 4-coumarate:CoA ligase; C3H, *p*-coumarate 3-hydroxylase; C4H, cinnamate 4-hydroxylase; CCoAOMT, caffeoyl-CoA O-methyltransferase; HCT, *p*-hydroxycinnamoyl-CoA:quinate shikimate *p*-hydroxycinnamoyltransferase; F5H, ferulate 5-hydroxylase; PAL, phenylalanine ammonia-lyase; SAD, sinapyl alcohol dehydrogenase. Compounds marked in red were downregulated in hpCCR1-1 lines. Those marked in blue were upregulated in hpCCR1-1 lines.

Over last year’s research in our group was focused on (i) functional analysis of the lignin specific genes in C3 and C4 grasses; (ii) understanding the roles, and substrate promiscuity, of catalytic enzymes of COMT family in shaping the composition of lignin polymers; and (iii) metabolic reprogramming of lignin pathway redirecting metabolic fluxes between S and G- pathways (changing S/G ratio) and between lignin and flavonoid pathways under reverse genetics conditions (Louie et al., 2010; Tu et al., 2010; Forster et al., 2013; Giordano et al., 2014a,b). Downregulation of *LpCCR1,* the first lignin-specific gene in *Lolium perenne* resulted in a decreased level of the downstream products in the lignin biosynthesis pathway, such as H, G, and S units, caffeoyl aldehyde, and coniferaldehyde (these compounds are shown in red in **Figure 5**). This suggests that coumaroyl-CoA and caffeoyl-CoA, along with feruloyl CoA, may serve as the primary substrates for CCR1 in perennial ryegrass. This also indicates that coniferaldehyde, a common intermediate of both G- and S-lignin biosynthesis, may be synthesized from feruloyl-CoA or from caffeyl aldehyde. The latter scenario correlates with predicted roles of LpOMT1 in methylation of 3-OH in caffeoyl aldehyde, suggesting an additional, caffeoyl aldehyde-mediated branch in both G- and S-related monolignol biosynthesis (Louie et al., 2010; Tu et al., 2010; **Figure 5**).

Molecular and metabolic cross-talks through redirection of metabolic fluxes between lignin and flavonoid pathways were shown for a variety of species with down regulated genes involved in phenylpropanoid, lignin, and flavonoid pathways. Silencing of the *CCR* gene in tobacco, tomato, and poplar results in decreased flux from feruloyl-CoA to G and S units reducing the level of lignin-specific phenolic molecules (van der Rest et al., 2006; Dauwe et al., 2007; Leple et al., 2007). By contrast, the levels and composition of the some of the stress-related flavonoid intermediates and derivatives were strongly enhanced in these transgenic lines. Most of the accumulating flavonoid metabolites are modified through quination and glucosylation by *p*-hydroxycinnamoyl-CoA:D quinate and a family of glycosyl transferases, respectively (van der Rest et al., 2006; Dauwe et al., 2007; Leple et al., 2007). Some molecules, such as coumaroyl-CoA, can be also conjugated with shikimate producing *p*-coumaroyl-shikimate by *p*-hydroxycinnamoyl-CoA:shikimate *p*-hydroxycinnamoyltransferase (**Figure 5**; Boerjan et al., 2003; Hoffmann et al., 2004; Dauwe et al., 2007).

Reduced flux from coumaroyl-CoA, caffeoyl-CoA, and feruloyl-CoA to H, G, and S units, as a result of downregulation of *LpCCR1* in *L. perenne* led to enhanced accumulation of coumaric acid, cinnamic acid, and ferulic acid (Tu et al., 2010). As a result, an elevation in the levels of *p*-coumaroyl quinic acid, caffeoyl quinic acid, and caffeoyl shikimic acid were occurring in hpCCR1-1 plants. Accumulated caffeoyl shikimate and caffeoyl quinate can be stored or, alternatively, converted back into caffeoyl-CoA and feruloyl-CoA (Schoch et al., 2001). Deficiency of CCR1 activity also diverts coumaroyl CoA esters into the flavonoid biosynthetic pathway, significantly increasing level of some of its components: coumaroyl quinic acid (up 14.1-fold), caffeoyl shikimic acid (up 5.9-fold), caffeoyl quinic acid (14.1-fold), rutin-7-glycoside (up 5.1-fold), kaempferol-*O-*rutinoside (up 4.3-fold), kaempferol-7,3-glycoside ferulic acid (up 3.9-fold), kaempferol diglucoside malonate (up 3.6-fold), rutin (up 3.1-fold), and kaempferol-*O*-glycoside (up 2.9-fold; Tu et al., 2010). Dramatic decrease in the levels of total lignin and S, G, and H subunits associated with enhanced levels of flavonol glycosides and acylated anthocyanins was shown for poplar, tobacco, and tomato transgenic plants (van der Rest et al., 2006; Besseau et al., 2007; Dauwe et al., 2007; Leple et al., 2007). Consequent modification of accumulated flavonols through glycosylation and quinylation in these plants enhanced detoxiflcation of the phenylpropanoid intermediates. Marles et al. (2003) and Marles and Gruber (2004) found that reduced lignin content is strongly associated with the changes in pigmentation of seed coat traits in the species of *Brassicaceae* representatives. The silencing of a lignin biosynthetic gene *hydroxycinnamoyl-CoA shikimate/quinate hydroxycinnamoyl transferase* in *A. thaliana* led to both, lignin synthesis repression and the redirection of the metabolic flux into flavonoids increasing CHS activity in transgenic plants (Besseau et al., 2007). Reverse genetics approach was successfully implemented also to show redirection of the carbon flow from lignin to cellulose. Downregulation of *4-coumarate-CoA ligase* in aspen (*Populus tremuloides*) resulted in a 45% decrease in lignin content and a concomitant 15% increase in cellulose content (Hu et al., 1999). These figures were further increased to a 52% reduction in lignin content and a 30% increase in cellulose content when coniferaldehyde 5-hydroxylase was also down-regulated (Li et al., 2003). Interaction between MYB75 with another secondary cell wall regulator, the KNOX transcription factor KNAT7, contributes to the regulation of secondary cell wall deposition in the *Arabidopsis* inflorescence stem re-directing the metabolic flux between the lignin, fiavonoid, and polysaccharide pathways (Bhargava et al., 2010). Down-regulation of another member of MYB-family in *Zea mays* reduced expression of several genes involved in the synthesis of monolignols, which significantly reduced lignin content in transgenic plants (Fornale et al., 2010, 2014). These plants also showed induced expression of stress-related proteins, suggesting redirection of carbon flux towards the biosynthesis of flavonoids.

Redirection of metabolic fluxes in opposite direction – from flavonoid into lignin pathway was observed in genetically modified strawberry (*Fragaria* × *ananassa*). Down-regulation of the members of *CHS* diverted the carbon flux from anthocyanin precursors into lignin pathway triggering accumulation of 4-coumaroyl-CoA-derived metabolites, such as 4-coumaryl alcohol and 4-coumaryl acetate (Brewer et al., 1985; Lunkenbein et al., 2006a,b). Simultaneous overexpression of *eugenol synthase* showed the enhanced production of the phenolic volatiles (Hoffmann et al., 2006, 2011). Similar results were shown recently by Ring et al. (2013) who showed that silencing of *CHS* in strawberry leads to the loss of pigmentation, which was accompanied by a significant increase in lignin content and, in turn, with enhanced firmness of the fruits.

## FLAVONOIDS AS NUTRACEUTICALS

Longer average life span, decreasing physical activity, and most importantly, the increasing consumption of unhealthy foods have been identified by the World Health Organization as the main triggers of chronic disease in the decade 2005–2015 (WHO, 2005). Plant biochemistry and metabolomics have significantly contributed to the wellbeing of human and animal health through identifying and understanding the health-promoting components of food, known as nutraceuticals (Hu and Willett, 2002; Espín et al., 2007; Rao and Rao, 2007; World Cancer Research Fund, 2007; Martin et al., 2011, 2013). The best-described property of almost every group of flavonoids is their capacity to act as antioxidants protecting the body against ROS (Tapas et al., 2008; Santhosh and Suriyanarayanan, 2014). The antioxidant activities (TEAC) per 100 g fresh weight, uncooked and portion size, are: strawberry >> raspberry = red plum >> red cabbage >>> grapefruit = orange > spinach > broccoli > green grape > onion > green cabbage > pea > apple > cauliflower > tomato peach = leek > banana = lettuce (Proteggente et al., 2002). Common dietary flavan-3-ols present in fresh fruits, vegetables, and nuts are catechin, catechin gallate, epicatechin, epicatechin gallate, epigallocatechin, epigallocatechin gallate, gallocatechin, and gallocatechin gallate. Dietary flavonoids also include the anthocyanins cyanidin, delphinidin, malvidin, pelargonidin, peonidin and petunidin together with the flavanones hesperetin and naringenin, the flavones apigenin and luteolin, and the flavonols myricetin, kaempferol, and quercetin (Grotewold, 2006b). Apart from antioxidant activities, food-derived flavonoids were shown to reduce the incidence of atherosclerosis (Terao, 2009), cancer (Clere et al., 2011), cardiovascular diseases (Bojić, 2011), diabetes (Zheng et al., 2011), thrombosis (Phang et al., 2011), inflammation in arthritis (Gonzalez et al., 2011), neurodegenerative diseases such as Alzheimer’s and Parkinson’s diseases (Mandel et al., 2011), obesity (Birari et al., 2011), hyperlipidemia (Wang et al., 2011), nerve injury (Jäger and Saaby, 2011) and hypertension (Cassidy et al., 2011).

The beneficial effects of proanthocyanidins on animal health and farm production have been extensively investigated. One of the most beneficial traits of important forage legumes is the level and composition of proanthocyanidins in their edible parts, leaves and stems. Ruminants fed on forages containing moderate amounts of proanthocyanidins show reduced methane gas emission, which decreases the chances of a digestive disorder, known as bloat. Bloat occurs when grazing ruminants consume large quantities of leguminous plants (e.g., alfalfa or clover). The gases produced in the rumen during fermentation cannot be released in the normal way since they are trapped in the foam caused by the rapid release of soluble proteins during chewing and ruminal degradation. Low concentrations of proanthocyanidins (around 5 mg/g dry matter) can be beneficial, reducing protein fermentation to ammonia in the rumen and methane gas emissions (Marles et al., 2003; Min et al., 2006). The mechanisms by which tannins reduce ruminal degradation of different dietary components are not entirely clear. Among the most accepted are substrate privation (McMahon et al., 2000), enzyme inhibition (Jones et al., 1994) and direct action on rumen microorganisms (Leinmüller et al., 1991). With respect to milk production, Wang et al. (1996a) reported an increase of 21% during mid and late lactation in sheep fed *Lotus corniculatus* (44.5 g CT kg^-1^ DM). They, and others, also reported significant increases in the efficiency of milk production, increased protein and lactose production, and a decrease in the fat content of the milk (Frutos et al., 2004). Enhanced amounts of foliar condensed tannins (2–4%) would also significantly contribute to forage quality via increased animal health and production and decreases in adverse environmental impacts.

Representatives of *Lotus* species, *L. pedunculatus, L. corniculatus* and *L. tenuis* contain up to 7.2, 4.6, and 0.8% of dry matter of proanthocyanidins in their leaves, respectively (Sivakumaran et al., 2006) with *L. corniculatus* containing the most desirable concentration for feeding ruminants (Miller and Ehlke, 1997; Escaray et al., 2012). In spite of their low concentration of proanthocyanidins, *L. tenuis* varieties are more tolerant to waterlogging, alkaline and salt conditions than any commercial varieties of *L. corniculatus.* Escaray et al. (2014) crossed a *L. tenuis* cultivar with a diploid, wild accession of *L. corniculatus* rich in proanthocyanidins and the *L. tenuis × L. corniculatus* F1 hybrid population obtained showed successful introgression of several traits from both parents, including the optimal concentration of proanthocyanidins in the herbage. Enhanced levels of proanthocyanidins in the hybrids correlated with increased expression levels of the R2R3MYB transcription factor *TT2* and a number of the key structural genes involved in biosynthesis of the building blocks of proanthocyanidins, epicatechin, and catechin. This exciting result highlights the importance of wild relatives as donor of useful traits and the power of traditional breeding to produce genetic pool for breeding Lotus varieties with superior nutritional value.

*Medicago sativa and T. repens,* the predominant pasture legume species contain no or very low levels of *proanthocyanidins* in foliar tissues (Hancock et al., 2014). Only two *Trifolium* species, namely *T. arvense* and *T. aﬄne,* are known to accumulate significant levels of proanthocyanidins in leaves (Fay and Dale, 1993). Screening of the cDNA library from *T. arvense* led to the isolation of the family of R2R3-MYB transcription factors. Silencing of *TaMYB14* resulted in almost complete cessation of proanthocyanidin biosynthesis in *T. arvense* (Hancock et al., 2012, 2014). Ectopic expression of *TaMYB14* in *M. sativa* and *T. repens* led to accumulation of proanthocyanidins in leaves up to 1.8% dry matter.

## ENGINEERING OF FLAVONOID PATHWAY

Recent progresses in genomics and functional genomics of flavonoid genes, coupled to extensive metabolomic studies, led to the development of efficient molecular tools to obtain crops with balance levels of flavonoids and related value products beneficial to human and animal health and for the exploitation of crops for the production of renewable energy. In general, engineering of flavonoid pathways in plants can be achieved using three commonly used strategies: (i) up-regulation of endogenous genes (enzymes and transcription factors), directing carbon flow toward the target products; (ii) down-regulation of the genes involved in competing pathways, and (iii) expression of exogenous genes targeting intermediate molecules towards biosynthesis of new products. Strategies to improve market acceptance of transgenic crops include elimination of sequences from non-plant origins and the generation of selectable marker-free events through co-expression of the genes of interest and selectable markers (Giordano et al., 2014b).

**FIGURE 6:**
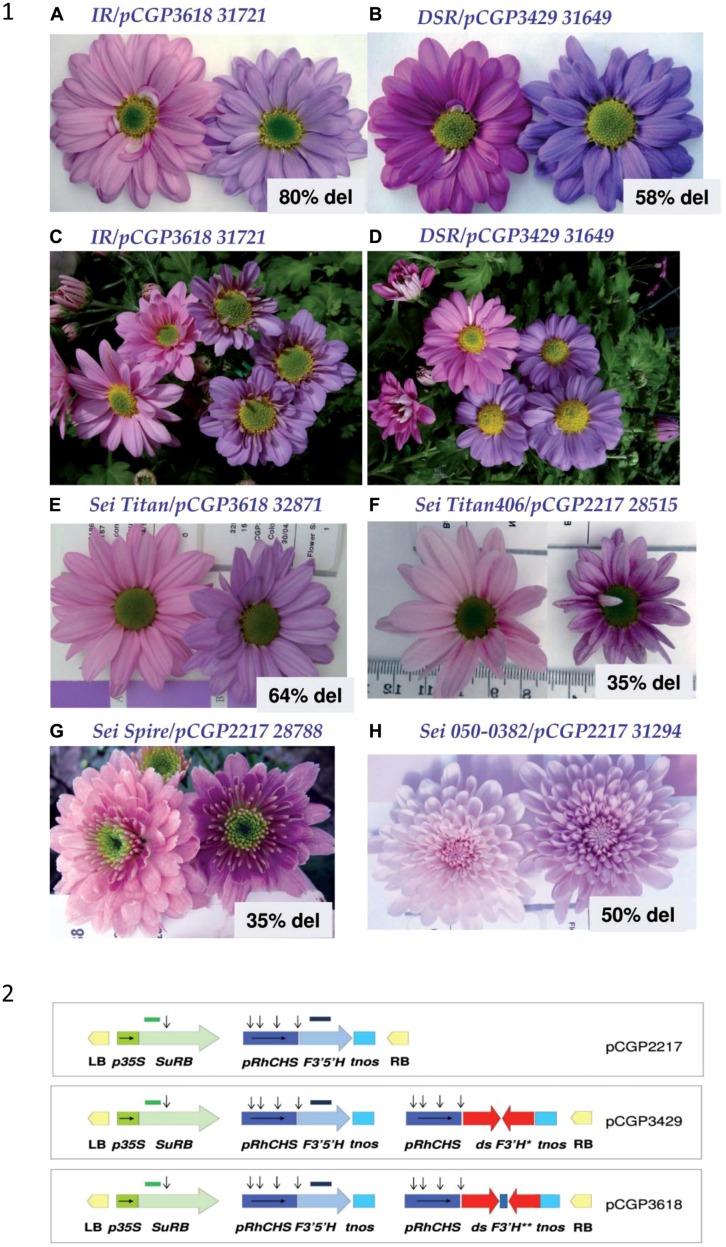
**Inflorescence color changes with the production of delphinidin-based anthocyanins (Brugliera et al., 2013).** (1; **A–H**) The host is on the left and the transgenic on the right. The percentage of delphinidin (of total anthocyanidins) detected in hydrolyzed petal extracts is given under the transgenic inflorescence. The chrysanthemum cultivars: IR, Improved Reagan; DSR, Dark Splendid Reagan; Sei; Sei Titan; Sei Spire. The transgenic line number is given next to the cultivar/construct. (2) Schematic representation of the T-DNA components of selected binary plasmid vectors used for plant transformation.

The engineering of the flavonoid pathway started more than 20 years ago with modification of anthocyanins level and composition in ornamental flowers through expression and/or silencing of the key flavonoid genes, *CHS, F3H, F3′5′H, DFR* (Meyer et al., 1987; Nakamura et al., 2006; Tanaka et al., 2008; Nishihara and Nakatsuka, 2011). Today a list of ornamental plants with modified floral colors include petunias, gerberas, roses, carnations, lisianthus, lotus, chrysanthemums, and torenia (Tanaka and Ohmiya, 2008; Tanaka et al., 2008, 2009; Nishihara and Nakatsuka, 2011; Brugliera et al., 2013). Most recent papers in this area describe reprogramming of the anthocyanin pathway in transgenic chrysanthemum and camellia plants (Brugliera et al., 2013; Zhou et al., 2013). Enhanced production of delphinidins was shown in floral petals of chrysanthemum after expression of the *F3′5′H* gene from pansy regulated by CHS promoter from rose (Brugliera et al., 2013). The additional silencing the endogenous *F3′H* gene resulted in more intensive blue coloration of floral petals (**Figure 6**). Expression of flavonol synthase from yellow flower of *Camellia nitidissima*, in *N. tabacum* altered floral color into white or light yellow, and metabolic analysis showed significant increasing of flavonols and reducing of anthocyanins in transgenic plants (Zhou et al., 2013). Shelf-life is one of the most important agronomic traits for tomato (*Solanum lycopersicum*) and is determined by their susceptibility to opportunistic pathogens, *Botrytis cinerea*. Over-production of anthocyanins in skins of genetically modified tomato was sufficient to reduce the susceptibility of fruit to *B. cinerea* (Bassolino et al., 2013).

Unexpected link between distant flavonoid and terpenoid pathway was recently shown when expression of the wild-type *CHI* gene complemented not only anthocyanin deficiency in *anthocyanin free* (*af*) tomato mutant but also terpenoid production in glandular trichomes. (Kang et al., 2014). These can be explained by the fact that these pathways have obvious complementary roles in plants protecting them from insect herbivores and microbial pathogens as well as mediating plant communication with symbiotic bacteria, natural enemies of arthropod herbivores, and parasitic plants (Hirsch et al., 2003; Rasmann et al., 2005).

Plant genetics and metabolic engineering have been extensively used to make human foods that differ in their content of specific and healthy phytonutrients (designer food). Recently, different transgenic strategies have been taken to increase flavonoid levels in tomato fruit by overexpressing either the heterologous structural or regulatory genes involved in the biosynthetic pathway (Bovy et al., 2002; Verhoeyen et al., 2002; Litster and Atwell, 2006; Luo et al., 2008; Zuluaga et al., 2008). Expression of two transcription factors from snapdragon, a bHLH-type (*Delila*) and R2R3 MYB-type (*Rosea1*) under the control of a fruit-specific promoter in tomato (cv. MicroTom) showed the strong visible purple pigmentation with the final concentration of anthocyanins averaged 3 mg/g fresh weight (Butelli et al., 2008), which is the highest value obtained so far in this species. In these plants expression of *Delila* and *Rosea1* has stimulated the transcription of cascade of the structural genes involved in the biosynthetic pathway, including *PAL, CHI* and *F3′5′H* directing flavonoid intermediates towards the anthocyanin products. A pilot animal test with a group of cancer susceptible mice that were fed with transgenic purple tomatoes showed a significant extension of their average life span compared with cancer-susceptible mice. This demonstrates beneficial role of these anthocyanin-rich tomatoes in human health. Other researches also showed up to 100-fold increase in anthocyanin concentration in transgenic tomato with up-regulated anthocyanin pathway (Maligeppagol et al., 2013).

Another example of engineering the flavonoid pathway to boost the accumulation of metabolites of pharmacological value for human health includes the modification of the pathway of the isoflavonoid phenolic acids. The production of isoflavones is restricted almost exclusively to the family *Fabaceae*. These metabolites are known to exhibit anticancerous and anti-osteoporotic activities in animal systems. Jiang et al. (2014) enhanced isoflavone production in soybean hairy roots by down-regulating two flavonoid genes, the F3H and the flavones synthase II^′′^. In tobacco, Pandey et al. (2014) showed a substantial amount of genistein glycoconjugates as a result of the co-expression of the transcription factor *AtMYB12* with the soybean *IFS* gene”. The rare wild species of snow lotus, *Saussurea involucrata* is a commonly used medicinal herb with great pharmacological value for human health, resulting from its uniquely high level of phenylpropanoid compound production. Transgenic *S. involucrata* co-expressing *PAP1* and *Lc* genes showed activation of most of the phenylpropanoid pathway genes, and increased accumulation of chlorogenic acid, syringin, cyanrine, and rutin greatly increasing its antioxidant capacity (Qiu et al., 2013).

Stilbenes play the important protective roles as antifungal phytoalexins and phytoanticipins and in mitigating UV-induced damages. The biosynthetic pathway for stilbenes diverges from the general phenylpropanoid pathway through the action of stilbene synthases, utilizing *p*-coumaroyl-CoA, and cinnamoyl-CoA starter units for the generation of the parent stilbene resveratrol and pinosylvin, respectively. Redirection of flavonoid pathway toward production of the stilbenes through co-expression of *stilbene O-methyltransferase* with a *stilbene synthase* genes from peanut in tobacco and *Arabidopsis* plants, resulted in the accumulation of pterostilbene in both species (Rimando et al., 2012). A reduced floral pigmentation phenotype observed in tobacco because of substantial decreases in both dihydroquercetin-derived flavonoids and phenylpropanoid-conjugated confirmed redirection of flavonoid intermediates into stilbene biosynthesis.

## CONCLUSION

Systemic biological approaches, combining genomics, proteomics, and metabolomics of flavonoids in plants, have provided new insights into the understanding of the origins of flavonoid biosynthesis and how they have evolved historically from Earth’s beginnings into one of the most widely spread pathways in modern plants. Recent progress in engineering of flavonoid pathways offers potential breakthroughs in the modification of secondary metabolite pathways of plants in ways not possible through conventional plant breeding. Remarkable plasticity of plant secondary metabolism could be exploited for the production of novel high-value pharmaceutical compounds not detected in wild type plants. Ability of flavonoids to evolve and modify constantly in response to new stressful environments was shown in response to ozone depletion that has become a major environmental problem over the last three decades (Rozema et al., 1997; Burchard et al., 2000). Stress-inducing generation of more and more sophisticated cascades of protecting molecules will keep flavonoids on the top of most important plant metabolites in 21 century and beyond.

## Conflict of Interest Statement

The authors declare that the research was conducted in the absence of any commercial or financial relationships that could be construed as a potential conflict of interest.
